# Task-Oriented Evaluation of the Feasible Kinematic Directional Capabilities for Robot Machining

**DOI:** 10.3390/s22114267

**Published:** 2022-06-03

**Authors:** Saša Stradovnik, Aleš Hace

**Affiliations:** Institute of Robotics, Faculty of Electrical Engineering and Computer Science, University of Maribor, 2000 Maribor, Slovenia; sasa.stradovnik@um.si

**Keywords:** robot surface machining, task feasibility, task-dependent kinematic capability, kinematic performance evaluation, manipulability index, manipulability polytope, motion decomposition, Decomposed Twist Feasibility (DTF) method

## Abstract

Performing the machining of complex surfaces can be a challenging task for a robot, especially in terms of collaborative robotics, where the available motion capabilities are greatly reduced in comparison with conventional industrial robot arms. It is necessary to evaluate these capabilities prior to task execution, for which we need efficient algorithms, especially in the case of flexible robot applications. To provide accurate and physically consistent information about the maximum kinematic capabilities while considering the requirements of the task, an approach called the Decomposed Twist Feasibility (DTF) method is proposed in this study. The evaluation of the maximum feasible end-effector velocity is based on the idea of decomposition into the linear and angular motion capabilities, considering a typical robot machining task with synchronous linear and angular motion. The proposed DTF method is presented by the well-known manipulability polytope concept. Unlike the existing methods that estimate the kinematic performance capabilities in arbitrarily weighted twist space, or separately in the translation and the rotation subspace, our approach offers an accurate and simple solution for the determination of the total kinematic performance capabilities, which is often highly required, especially in the case of robot machining tasks. The numerical results obtained in this study show the effectiveness of the proposed approach. Moreover, the proposed DTF method could represent suitable kinematic performance criteria for the optimal placement of predefined tasks within the robot workspace.

## 1. Introduction

Collaborative robotic technologies enable humans and robots to work close together in a shared manufacturing environment. Contrary to classical industrial robots, which are primarily programmed to perform deterministic repetitive tasks, a new paradigm of collaborative robots encourages the development of flexible and intelligent robotic systems, especially for the needs of small and medium enterprises with low-volume, high-mix production [1,2]. In order to help these companies to gain a competitive advantage, a rapid integration into the manufacturing process is needed in order for them to achieve improved flexibility, higher autonomy, and safety of a robotic system for the industrial floor. In this context, recent studies on intuitive motion planning algorithms [3,4] and strategies toward safe and efficient human–robot collaboration [5,6] have attracted growing attention. However, in order to further improve flexibility and the easy deployment of collaborative robots, efficient trajectory planning algorithms for complex robot machining tasks are also necessary [7]. To provide autonomous and singularity-free motion planning, a detailed understanding of available motion capabilities within a robot’s workspace is key for the successful execution of complex robot machining tasks [8].

A robot’s workspace is usually defined as the maximum reachable volume whereby the end-effector is capable of reaching each point in at least one orientation. The reachable workspace characteristics can be computed offline and visualized as a discretized map, which is called a reachability or a capability map [9,10]. The manipulability of the robotic mechanism near the singular position can be largely reduced, although the robot can reach each point within a dexterous workspace—which is the subset of the reachable workspace—with any orientation. Consequently, there is still no guarantee for a successful and singularity-free Cartesian movement between two reachable poses in an operational task space. Therefore, kinematic and dynamic constraints such as positional joint limits, joint velocity, acceleration, and torque limits should also be considered when planning a feasible robotic task, for which the required kinematic and dynamic capabilities should be lower than the maximum allowable values of the robot.

A detailed understanding of robot movement capability within its workspace is crucial for the successful planning and execution of more complex surface machining tasks that require the simultaneous tracking of the desired workpiece surface all the time along the path (robotic milling, welding, composite layup, hammer peening, etc.) [11,12,13,14]. The execution of the complex robot task is not necessarily feasible anywhere in the robot workspace, especially in the case of collaborative robots, where the margin between their joint capabilities and task requirements is greatly reduced compared to conventional industrial robots [15]. To determine the optimal placement of a robot task within the robot workspace, it is necessary to propose a suitable objective function for optimization. In order to optimize a robotic machining trajectory, some existing kinematic performance indices are often taken into consideration [16,17,18]. A detailed analysis of these methods has shown that the most of them suffer from physical inconsistency (inhomogeneous Jacobian matrix [19], metric properties of twist [20]), do not take exact task requirements into account (such as task direction, synchronous linear and angular motion), do not include maximum kinematic constraints into consideration, or they are computationally intensive.

If only the kinematic of the robot is considered, a qualitative measure of the ability to move the end-effector toward an arbitrary direction is most often based on analyzing the manipulability ellipsoid that was first defined by Yoshikawa [21]. Yoshikawa’s manipulability index is proportional to the volume of the manipulability ellipsoid and should give information about the proximity to a singular configuration, which can also be estimated by the condition number or minimal singular value [22]. These indices can provide general evaluations of the robot’s capabilities in order to optimize the design of the robot [23] or any other global characteristic of a robot. However, such performance measures are often of limited practical usefulness since they are not reliable enough if the directional kinematic capabilities have to be improved along the predefined robotic task.

A suitable performance metric to determine the maximum directional kinematic capabilities of the robot end-effector was proposed in [24], that being the vector length from the center of the manipulability ellipsoid to the surface intersection point in the desired direction. It is also frequently referred to as the manipulator velocity ratio, first introduced in [25]. An alternative approach has been proposed based on the manipulability polytope [26], which is derived from the exact joint velocity limits. The ellipsoid approach, which involves the Euclidian norm, can provide an estimation of a robot’s capabilities in a task space relative to the effort in a joint space. However, the manipulability polytope appears to be more appropriate for the estimation of the robot’s feasible capabilities in the task space constrained by the joint velocity limits.

Both methods may result in a combination of translational and angular motion capabilities in a single scalar performance measure, which makes them physically inconsistent [27]. Several approaches have been suggested to overcome the drawback of physical inconsistency. One of the most common weights the translation capacity against the rotational [25], or handles them in a separate manner [28]. Different methods exist for the determination of the weights. However, arbitrariness in the choice of the weights is unavoidable [29]. The choice is usually made based on the task specifications [30] or based on the minimal principal axes of the translational and rotational subspace [31]. Some authors attempt to avoid this problem by introducing the new performance indices [32,33]; however, they only include translational directional capacities in their consideration. A new approach based on power manipulability and considered to be fully homogeneous and physically consistent has also been proposed in [34], but a direct relation between available power and specific task requirements could not be established clearly.

The more objective approach seems to be the partition on the translational and rotational motion capabilities. Through the decomposition of the task space into a translational and rotational subspace, the translational and rotational manipulability polytope in a weak and strong sense was derived in [35], similarly to Yoshikawa’s approach [36] in the case of the manipulability ellipsoids. When the translational (rotational) manipulability polytope in a weak sense is taken into consideration, the maximum possible linear (angular) velocity can be evaluated separately from the rotational (translational) subspace, whereby the angular (linear) velocity may not be zero and possibly uncontrolled. That means that the length of the translational (rotational) manipulability polytope represents the maximal feasible linear (angular) velocity in the specified direction, generating a simultaneous movement in the rotational (translational) subspace, which cannot be arbitrarily selected in accordance with the task requirements. Thus, this approach can be useful only for the applications where the angular velocity is irrelevant to the task performance [28,35,37]. However, in the case of robot machining applications, the desired synchronization between both subspaces according to the task requirements must be taken into account. Only pure translational (rotational) motion can be analyzed by the translational (rotational) manipulability polytope in a strong sense [36,38,39], but such applications, especially in the case of the robot surface machining, rarely exist in practical industrial cases. Some more recent approaches have evaluated the maximum directional kinematic capability using optimization-based methods in the case of redundant manipulators [40,41], which are mostly used in the case of redundant robot manipulators. If suitable linear inequality constrains related to the specific task requirements can be defined, the formulation of the optimization problem can also be adopted for robot machining purposes. However, finding the solution in this way is a time-consuming process. Generally, none of the above-mentioned methods seem to be suitable nor optimal, nor do any of them involve an appropriate consideration of the scenario when synchronized translational and rotational motion is required.

For this reason, we propose a method called Decomposed Twist Feasibility (DTF) in order to determine a feasible linear and angular velocity for robot machining purposes. In contrast to the existing methods that estimate maximum directional capabilities, it can precisely determine maximum motion capabilities for translation and rotation while considering the exact joint velocity limits and specific task requirements. Physical consistency can be achieved since the translational and rotational performance capabilities are considered individually. In contrast to the polytope approach, our solution can be computed in a time-efficient manner. The test results based on a generic 3 DOF planar robot manipulator and a collaborative robot UR5e demonstrate the usefulness of the proposed DTF method. 

The remainder of this article is organized as follows. The related research based on the manipulability ellipsoid and polytope is described in Section 2. In this section, we also introduce a DTF method to determine the feasible linear and angular velocity in synchronous motion, which is required in applications such as robot surface machining. In Section 3, the numerical test results of the proposed DTF method in the case of a generic 3 DOF planar robot mechanism and a collaborative robot UR5e are shown in the context of the twist manipulability polytope, which proves the effectiveness and usefulness of the proposed DTF method. Finally, a discussion with conclusions is given in Section 4.

## 2. Materials and Methods

### 2.1. Manipulability Ellipsoid

The forward velocity kinematics of an *n* degree-of-freedom serial robotic manipulator, which we consider to be operating in an *m*-dimensional Euclidian operational space, describes the relation between the end-effector velocities *t*, called twist, and joint velocities $\dot{\theta }$:

(1) $$t=\left[\begin{array}{c} v\\ \omega \end{array}\right]=J(\theta )\dot{\theta },$$

where t is defined as a set of the linear velocity vector $v\in {\mathbb{R}}^{3}$ and the angular velocity vector $\omega \in {\mathbb{R}}^{3}$; $J(\theta )\in {\mathbb{R}}^{mxn}$ is a Jacobian matrix of the manipulator, with components of $J_{ij}=\partial f_{i}(\theta )/\partial \theta_{j}$, where *i* = 1…*m*, *j* = 1…*n*, and m=6; $\theta \in {\mathbb{R}}^{n}$ is the joint position vector, and $\dot{\theta }\in {\mathbb{R}}^{n}$ defines the joint velocity vector. We assume rotational joints without a significant loss of generality.

The most widely used kinematic performance measure, which is based on the Jacobian matrix, was introduced in [21]. The velocity manipulability ellipsoid of a given configuration provides an intuitive graphical representation of how efficient the mapping of velocities is from the joint space to the task-space. The set of joint velocities with the unit Euclidian norm associated with a unit sphere in the joint velocity space:

(2) $$E=\left\{\dot{\theta }\in {\mathbb{R}}^{n}\;|\;{\dot{\theta }}^{T}\dot{\theta }\leq 1\right\}$$

maps into the velocity manipulability ellipsoid ε in the task space:

(3) $$\varepsilon =\left\{t^{T}{\left(JJ^{T}\right)}^{-1}t\leq 1\right\}.$$

Note that ${\dot{\theta }}^{T}\dot{\theta }$ is equal to the square of the Euclidian norm ${\left\|\dot{\theta }\right\|}_{2}^{2}$ of a vector $\dot{\theta }$. The volume of ε, however, may be used to define the quantitative measure of manipulability w:

(4) $$w=\sqrt{\det(JJ^{T})},$$

which is often used, although it suffers from a few limitations related to the physical inconsistency of the Jacobian matrix [19]. Furthermore, despite its popularity, the manipulability measure only gives a rough estimation of the robot’s movement capability and closeness to the singularity, and it is not accurate enough to determine how fast the robot can move in a certain direction. The manipulability ellipsoid principal axes—the direction of which coincides with the eigenvectors of the square matrix $JJ^{T}$, and whose lengths correspond to the singular values of J—represent the best and worst directions for the robot to perform a movement. 

A robot’s movement capability in any other direction can be geometrically described as the distance from the center of the ellipsoid to the point where the line along the direction of interest intersects the ellipsoid surface. Let $\hat{u}$ denote the unit vector in the task velocity direction of interest, and $\mu_{2}$ be the distance from the ellipsoid center to the intersection point on the ellipsoid surface in the direction along the vector $\hat{u}$. Thus, one can write $t=\mu_{2}\hat{u}$, and in combination with (3), the following equation can be derived:

(5) $${\left(\mu_{2}\hat{u}\right)}^{T}{\left(JJ^{T}\right)}^{-1}(\mu_{2}\hat{u})=1.$$

The scalar $\mu_{2}$ is then the velocity transmission ratio in the direction of $\hat{u}$ introduced by Chiu [24]:

(6) $$\mu_{2}={\left[{\hat{u}}^{T}{\left(JJ^{T}\right)}^{-1}\hat{u}\right]}^{-1/2}.$$

The velocity transmission ratio (6), also known as the manipulator velocity ratio [25] or directional manipulability measure [42], can also be derived from the ratio of the task velocity vector norm to the joint velocity vector norm:

(7) $$\mu_{2}=\left\|t\right\|/\left\|\dot{\theta }\right\|.$$

The value of this index defines how effectively the robot’s end-effector can move in the direction $\hat{u}$ to satisfy condition ${\left\|\dot{\theta }\right\|}_{2}\leq 1$ in the joint space. The direction in which the velocity transmission ratio is at its maximum is the optimal task direction for affecting velocity, as the lowest robot overall kinematic effort is needed. However, since a natural norm does not exist in the space used to represent the twist [29], which involves both linear and angular velocities, it leads to the physically non-consistent definition of the performance index with no clear physical meaning [19].

### 2.2. Manipulability Polytope

The main disadvantage of the manipulability ellipsoid is the fact that it does not transform the exact joint velocity constraints into the task space [43]; however, it rather serves as a conservative approximation of the end-effector velocity capabilities since it relies on the Euclidean norm. In practical cases, the overall kinematic performance of the end-effector in a task space is bounded by each joint velocity limit [26]:

(8) $$\max\left|{\dot{\theta }}_{i}\right|\leq 1,$$

where ${\dot{\theta }}_{i}$ denotes the joint velocity of the *i*-th joint if we assume that the maximal joint velocity is limited by the unit value of 1. The bounds on the individual joint velocity form a convex joint velocity polytope Q with $2^{n}$ vertices in the *n*-dimensional joint space, which can be written in the following compact form:

(9) $$Q=\left\{\dot{\theta }\in {\mathbb{R}}^{n}\;|\;{\left\|\dot{\theta }\right\|}_{\infty }\leq 1\right\},$$

where ${\left\|.\right\|}_{\infty }$ denotes the infinity norm or $L_{\infty }$ norm, which is equal to the maximum absolute value of the vector’s components. The linear mapping of Q from the joint to the task space (1) results in another convex polytope $P^{twist}$ with the same number of vertices, which is called a twist manipulability polytope [15]:

(10) $$P^{twist}=\left\{t\in {\mathbb{R}}^{m}|\;t=J(\theta )\dot{\theta },\;{\left\|\dot{\theta }\right\|}_{\infty }\leq 1\right\}.$$

Since we are interested in quantifying the ability to change the position and orientation of the end-effector along a specified task direction, the maximum feasible magnitude of the end-effector velocity along $\hat{u}$ under the velocity constraints (8) can be defined as follows [28]:

(11) $$\mu_{\infty }={\left(\underset{i}{\max\left|{\dot{\theta }}_{i}^{*}\right|}\right)}^{-1},$$

where ${\dot{\theta }}^{*}={\left[{\dot{\theta }}_{1}^{*},{\dot{\theta }}_{2}^{*},\ldots ,{\dot{\theta }}_{n}^{*}\right]}^{T}$ is the minimum infinity norm solution of $\hat{u}=J\dot{\theta }$. The end-effector velocity (11) can be derived from the quotient of the weighted task velocity vector Euclidian norm and the joint velocity vector infinity norm [44]:

(12) $$\mu_{\infty }=\left\|t\right\|/{\left\|\dot{\theta }\right\|}_{\infty },$$

where the joint velocity vector $\dot{\theta }$ belongs to the input polytope Q (9), which is mapped into the output task space velocity polytope calculated by (1). Geometrically, $\mu_{\infty }$ is the distance from the center of the output polytope to the point where the line along the desired direction of $\hat{u}$ intersects the polytope.

Similar to the manipulability ellipsoid concept, the manipulability polytope concept also suffers from physical inconsistency due to the unwanted mixing of the linear and angular velocity in a single scalar value. Furthermore, it may be subjected to an algorithm seeking a computationally complex and less tractable minimum infinity norm solution [35,45,46].

### 2.3. Translational and Rotational Manipulability

Due to the physical inconsistency of the twist space manipulability ellipsoids and polytopes, the capabilities of translational and angular velocity should be handled individually. For this purpose, Yoshikawa [36] decomposed the task space into translational and rotational subspaces. He defined the translational (rotational) manipulability ellipsoid in the weak sense as a set of all translational velocities that are realizable under the constraint ${\left\|\dot{\theta }\right\|}_{2}\leq 1$. The translational (rotational) manipulability ellipsoid in the strong sense has an additional constraint, which requires the end-effector orientation to stay constant (angular velocity is zero, ω=0).

Similarly to the Yoshikawa approach, in the case of a manipulability ellipsoid, the translational and rotational velocity capabilities were also handled separately for the polytope approach [28]. Since the polytopes provide a more accurate estimation of the maximum achievable end-effector velocities compared to the ellipsoids, only decomposed manipulability polytopes will be considered in the following. In this case, the weak sense polytope may result in two different types, i.e., with the minimum $L_{\infty }$ norm and with the least $L_{2}$ norm solution of joint velocities, respectively. 

The translational (rotational) velocity polytope $P_{\infty }^{T}$($P_{\infty }^{R}$) in the $L_{\infty }$ weak sense is defined as the set of all linear (angular) velocities that are realizable under the constraints (8) with the minimum infinity norm solution, if the joint velocity polytope is transformed to the task space via $J_{T}$ ($J_{R}$):

(13) $$P_{\infty }^{T}=\left\{v\in {\mathbb{R}}^{3}|\;v=J_{T}\dot{\theta },\;{\left\|\dot{\theta }\right\|}_{\infty }\leq 1\right\}$$

(14) $$P_{\infty }^{R}=\left\{\omega \in {\mathbb{R}}^{3}|\;\omega =J_{R}\dot{\theta },\;{\left\|\dot{\theta }\right\|}_{\infty }\leq 1\right\}$$

where the Jacobian matrix is partitioned into translational and rotational (3x*n*) submatrices:

(15) $$J=\left[\begin{array}{c} J_{T}\\ J_{R} \end{array}\right]$$

**Figure 1:**
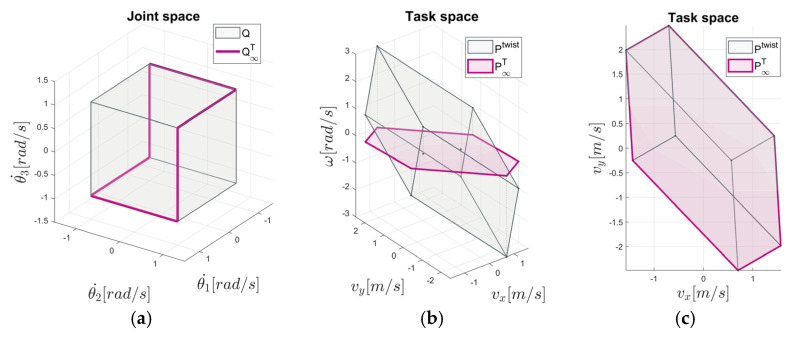
Example of a manipulability polytope representation based on the 3DOF planar manipulator: (**a**) Joint velocity polytope Q and the minimum $L_{\infty }$ norm solution of joint velocities represented by $Q_{\infty }^{T}$; the task space polytopes $P^{twist}$ and $P_{\infty }^{T}$ in the isometric view (**b**), and in the top view (**c**).

Note that mapping $v=J_{T}\dot{\theta }$ in (13) ($\omega =J_{R}\dot{\theta }$ in (14)) describes the under-determined linear system. The translational polytope $P_{\infty }^{T}$ is illustrated in Figure 1.

In Figure 1b,c, the example of a translational velocity polytope $P_{\infty }^{T}$ in comparison to the twist polytope $P^{twist}$ is presented in two different views in the task space. From Figure 1c, it can easily be seen that the translational velocity polytope $P_{\infty }^{T}$ fits to the orthogonal projection of the twist manipulability polytope $P^{twist}$ in the translational subspace.

When an *n*-dimensional polytope is mapped to a space of lower dimension (*k < n*), some of the vertices of the original polytope are mapped into an internal region of the *k-*dimensional polytope [47]. For this kind of redundancy, part of the joint velocities may produce end-effector velocities with non-zero rotation (translation) components. The maximum feasible linear end-effector velocity (which could be denoted by $v_{\infty }$) in a certain translational direction ${\hat{u}}_{T}\in {\mathbb{R}}^{3}$, or the maximum feasible angular end-effector velocity (which could be denoted by $\omega_{\infty }$) in the specific rotational direction ${\hat{u}}_{R}\in {\mathbb{R}}^{3}$, can be determined by geometry-based methods [43,45,48] as the vector length from the origin of the translational (rotational) velocity polytope $P_{\infty }^{T}$ ($P_{\infty }^{R}$) to the intersection with its boundary in the specified direction.

One may select one of the subspace manipulability polytopes, depending on the task at hand. If the main concern of the task is maximal linear velocity, then the vector length of the translational velocity polytope will be considered, and vice versa. Since translational subspace is considered separately from rotational subspace, multiple solutions may exist in the joint space, which satisfies constraint (8). The minimum infinity norm solution, illustrated by $Q_{\infty }^{T}$ in Figure 1a, can be found numerically based on the structured linear algebra algorithm [46], which is rather computationally extensive. Alternatively, this problem can be expressed as a linear programming problem, where the optimal solution exists at the intersection of the explicitly given linear inequality constraints.

Besides polytope analysis, performance indices based on the vector expansion method [49] offer a computationally faster solution compared to the polytopes approach, since only the robot’s capabilities are investigated in the direction of interest in the task space. The maximum achievable linear (angular) end-effector velocity in the ${\hat{u}}_{T}$ $\left({\hat{u}}_{R}\right)$ direction, based on the vector expansion method, can be estimated by the following:

(16) $$v_{2}={\left\|J_{T}^{†}{\hat{u}}_{T}\right\|}_{\infty }^{-1}$$

(17) $$\omega_{2}={\left\|J_{R}^{†}{\hat{u}}_{R}\right\|}_{\infty }^{-1}$$

The value $v_{2}$($\omega_{2}$) matches the vector length of a manipulability polytope $P_{2}^{T}$ ($P_{2}^{R}$) in the $L_{2}$ weak sense, with the least Euclidian norm solution of joint velocities in the direction defined by ${\hat{u}}_{T}$ (${\hat{u}}_{R}$). The polytope $P_{2}^{T}$ ($P_{2}^{R}$) is inscribed in the translational (rotational) manipulability polytope $P_{\infty }^{T}$ ($P_{\infty }^{R}$) in the $L_{2}$ weak sense, defined as follows:

(18) $$P_{2}^{T}=\left\{v\in {\mathbb{R}}^{3}|\;v=J_{T}\dot{\theta },\;{\left\|\dot{\theta }\right\|}_{\infty }\leq 1,\;\dot{\theta }=J_{T}^{†}v\right\}$$

(19) $$P_{2}^{R}=\left\{\omega \in {\mathbb{R}}^{3}|\;\omega =J_{R}\dot{\theta },\;{\left\|\dot{\theta }\right\|}_{\infty }\leq 1,\;\dot{\theta }=J_{R}^{†}\omega \right\}$$

which determines a set of joint velocities defined by the intersection $Q\cap Row(J_{T})$$\left(Q\cap Row(J_{R})\right)$ in the joint space, where $Row(J_{T})$ $\left(Row(J_{R})\right)$ is the row space of the translational (rotational) Jacobian matrix.

**Figure 2:**
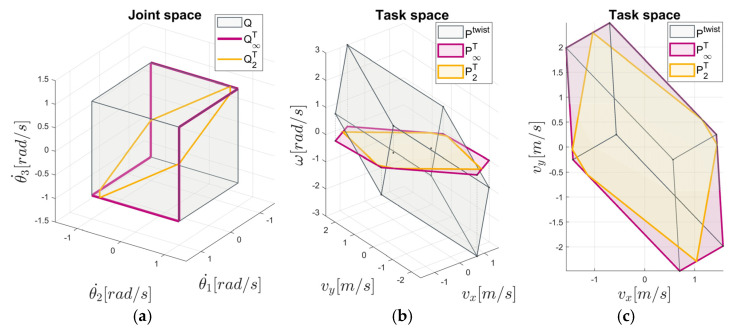
Example of a manipulability polytope representation based on the 3DOF planar manipulator: (**a**) Joint velocity polytope Q, the minimum $L_{\infty }$ norm solution of joint velocities represented by $Q_{\infty }^{T}$, and the least $L_{2}$ norm solution of joint velocities represented by $Q_{2}^{T}$; the task space polytopes $P^{twist},P_{\infty }^{T}$, $P_{\infty }^{T}$, and $P_{2}^{T}$ in the isometric view (**b**), and in the top view (**c**).

The polytope $P_{2}^{T}$ is depicted in Figure 2. In Figure 2b,c, the polytope $P_{2}^{T}$ in relation to the polytopes $P^{twist}$ and $P_{\infty }^{T}$ is shown in two different views in the task space, where it can be seen that $P_{2}^{T}$ is inscribed in the $P_{\infty }^{T}$. Unlike the polytope $P_{\infty }^{T}$, which defines the minimum $L_{\infty }$ norm solution of the joint velocities, the polytope $P_{2}^{T}$ represents the maximal linear velocities with the least $L_{2}$ norm solution of the joint velocities under the constraint (8), i.e., ${\left\|\dot{\theta }\right\|}_{\infty }\leq 1$, illustrated by $Q_{2}^{T}$ in Figure 2a.

The polytope $P_{2}^{T}$ is depicted by Figure 2. In Figure 2b,c, the polytope $P_{2}^{T}$ in relation to the polytopes $P^{twist}$ and $P_{\infty }^{T}$ is shown in two different views in the task space, where it can be seen that $P_{2}^{T}$ is inscribed in the $P_{\infty }^{T}$. Unlike the polytope $P_{\infty }^{T}$, which defines the minimum $L_{\infty }$ norm solution of the joint velocities, the polytope $P_{2}^{T}$ represents the maximal linear velocities with the least $L_{2}$ norm solution of the joint velocities under the constraint (8), i.e., ${\left\|\dot{\theta }\right\|}_{\infty }\leq 1$, illustrated by $Q_{2}^{T}$ in Figure 2a.

In terms of trajectory planning optimization, an analysis of the translational manipulability polytope in the $L_{2}$ or $L_{\infty }$ weak sense can offer a trajectory planning that reaches the goal pose faster [28] or with lower kinematic effort, assuming that the orientation of the end-effector is not important. However, how can translational capabilities be managed separately from the rotational and also keep control over a rotational subspace?

If we add another constraint that requires joint velocities to be projected onto the null space of $J_{R}$ ($J_{T}$), the translational (rotational) manipulability polytope in the strong sense can be obtained, which enables the analysis of purely translational (rotational) motions. In order to graphically represent the translational (rotational) manipulability polytope in the strong sense, it is necessary to find the intersection between the joint velocity polytope Q and the null space $N(J_{R})$$\left(N(J_{T})\right)$, i.e., $Q\cap N(J_{T})$ $\left(Q\cap N(J_{R})\right)$. The mapping of such joint velocities to the task space forms a translational (rotational) manipulability polytope in the strong sense $P_{strong}^{T}$$\left(P_{strong}^{R}\right)$, with the shape depending on the dimension of the considered null space:

(20) $$P_{strong}^{T}=\left\{v\in {\mathbb{R}}^{3}|\;v=J_{T}\dot{\theta },\;{\left\|\dot{\theta }\right\|}_{\infty }\leq 1,\;J_{R}\dot{\theta }=0\right\}$$

(21) $$P_{strong}^{R}=\left\{\omega \in {\mathbb{R}}^{3}|\;\omega =J_{R}\dot{\theta },\;{\left\|\dot{\theta }\right\|}_{\infty }\leq 1,\;J_{T}\dot{\theta }=0\right\}$$

**Figure 3:**
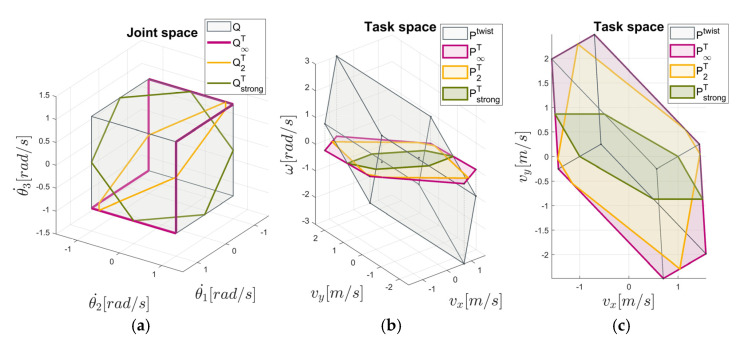
Example of a manipulability polytope representation based on the 3DOF planar manipulator: (**a**) Joint velocity polytope *Q*, the minimum $L_{\infty }$ norm solution of the joint velocities represented by $Q_{\infty }^{T}$, the least $L_{2}$ norm solution of the joint velocities represented by $Q_{2}^{T}$, and the intersection between the joint velocity polytope Q and the null space $N(J_{R})$ represented by $Q_{strong}^{T}$; the task space polytopes $P^{twist}$, $P_{\infty }^{T}$, $P_{2}^{T}$, and $P_{strong}^{T}$ in the isometric view (**b**) and in the top view (**c**).

The translational manipulability polytope $P_{strong}^{T}$ is depicted in Figure 3. A comparison with the polytopes $P^{twist}$, $P_{\infty }^{T}$, and $P_{2}^{T}$ is shown in Figure 3b,c. The polytope $P_{strong}^{T}$ is quite reduced in comparison to the polytopes $P_{\infty }^{T}$ and $P_{2}^{T}$. The joint velocity solution under the constraint $J_{R}\dot{\theta }=0$ and (8), i.e., ${\left\|\dot{\theta }\right\|}_{\infty }\leq 1$, is illustrated by $Q_{strong}^{T}$ in Figure 3a.

The translational (rotational) manipulability polytope in the strong sense $P_{strong}^{T}$ $\left(P_{strong}^{R}\right)$ allows for a complete separation of the translational and rotational capability analyses since only pure translational (rotational) movement is being considered at a time. What if a robot task requires a simultaneous motion with synchronized translational and rotational end-effector velocities, as in the case of robot surface machining? For this purpose, a solution will be derived in the following.

### 2.4. Proposed Method

The estimation of the maximum feasible linear and angular velocity for simultaneous linear and angular motion using the proposed DTF method will be explained in this section. The problem can be formulated as follows: We seek a maximum linear (angular) velocity in the desired direction, in a rotational (translational) motion synchronized with the specified direction, under joint velocity constraints (8). In the following, we assume an *n* degree-of-freedom, non-redundant, robotic serial-link manipulator in a non-singular configuration.

Unlike the majority of existing methods that take the translational (rotational) Jacobian submatrix $J_{T}$ ($J_{R}$) into consideration, the derivation of the proposed DTF method is based on the Jacobian submatrices, which fulfils the additional restrictions with the null space projection [36,38]. Let ${\tilde{J}}_{T}$ denote a 3x*n* translational Jacobian submatrix:

(22) $${\tilde{J}}_{T}=J_{T}(I-J_{R}^{†}J_{R}),$$

where I is the identity matrix, and $\left(I-J_{R}^{†}J_{R}\right)$ is the projector to the rotational null subspace, with the operator ${\left(.\right)}^{†}$ that stands for the Moore–Penrose pseudoinverse of a matrix. Similarly, the rotational Jacobian submatrix ${\tilde{J}}_{R}$ can be obtained. If we assume a square Jacobian matrix, then it can be shown that the inverse of the Jacobian (15) can be represented by two parts, which can be derived from the pseudoinverse of ${\tilde{J}}_{T}$ and ${\tilde{J}}_{R}$ [35,36]:

(23) $$J^{-1}=\left[\begin{array}{cc} {\tilde{J}}_{T}^{†} & {\tilde{J}}_{R}^{†} \end{array}\right],$$

such that the joint velocity vector can be decomposed correspondingly as:

(24) $$\dot{\theta }=J^{-1}\cdot t={\dot{\theta }}_{T}+{\dot{\theta }}_{R},$$

where ${\dot{\theta }}_{T}\in N(J_{R})$ and ${\dot{\theta }}_{R}\in N(J_{T})$. Note that in the latter equation, we assume that the joint velocity vector can be described in a form consisting of two parts related to translation and rotation. This can be rewritten as follows:

(25) $$\dot{\theta }={\tilde{J}}_{T}^{†}v+{\tilde{J}}_{R}^{†}\omega ,$$

where the first part of (25) are the joint velocities ${\dot{\theta }}_{T}\in N(J_{R})$ required solely by the translational end-effector displacement (i.e., with zero contribution to the rotational motion), while the second part are joint velocities ${\dot{\theta }}_{R}\in N(J_{T})$ responsible solely for rotational end-effector displacement (i.e., with zero contribution to the translational motion). To find the maximal velocity capabilities in the task space, we need to analyze how well the task’s requirements fit the robot’s joint velocity constraints (8). A robot surface machining path consists of waypoints located along a curved surface. During the process, the robot needs to follow the prescribed path with a constant linear velocity while maintaining the end-effector orientation normal to the curved surface. Synchronization between the desired linear and angular velocity vectors within two neighboring waypoints can be determined by the curved surface geometry.

In contrast to the existing methods, where the desired motion synchronization cannot be controlled (the maximum kinematic capabilities can be evaluated only based on the desired linear/angular velocity direction), the proposed method also takes the desired motion synchronization into consideration. The ratio of these magnitudes denoted by h∈ℝ defines the relative importance of the synchronized translational and rotational motion independently of the velocity profile scaling:

(26) h=V/Ω,

where $V={\left\|v\right\|}_{2}$ is the magnitude of the linear velocity, and $\Omega ={\left\|\omega \right\|}_{2}$ is the magnitude of the angular velocity. The parameter *h* is a task-dependent velocity ratio factor that reflects the synchronization of translational and rotational motion.

The linear velocity vector and the angular velocity vector can be represented by:

(27) $$v=V\cdot {\hat{u}}_{T},$$

(28) $$\omega =\Omega \cdot {\hat{u}}_{R},$$

where ${\hat{u}}_{T}\in {\mathbb{R}}^{3}$ and ${\hat{u}}_{R}\in {\mathbb{R}}^{3}$ are the unit vectors in the desired direction of the linear and the angular velocities in the task space, respectively. When the end-effector maintains a constant orientation, there is no rotational motion required (ω=0), thus the ratio h approaches infinity and the motion is purely linear. The maximum directional kinematic capabilities can be obtained by the translational manipulability polytope $P_{strong}^{T}$ in the strong sense. If the velocity ratio factor h is zero, the motion is pure rotation. The maximum directional kinematic capabilities can be determined by the rotational manipulability polytope $P_{strong}^{R}$ in the strong sense. Combining the task requirements and the robot’s velocity capabilities leads to the following:

(29) $${\left\|{\tilde{J}}_{T}^{†}V{\hat{u}}_{T}+{\tilde{J}}_{R}^{†}\Omega {\hat{u}}_{R}\right\|}_{\infty }\leq 1.$$

The maximum linear and angular velocity can be found by scaling under the constraints (8), considering (26). This yields the following equations:

(30) $$V_{\max}={\left\|{\tilde{J}}_{T}^{†}{\hat{u}}_{T}+h^{-1}\cdot {\tilde{J}}_{R}^{†}{\hat{u}}_{R}\right\|}_{\infty }^{-1},$$

(31) $$\Omega_{\max}={\left\|h\cdot {\tilde{J}}_{T}^{†}{\hat{u}}_{T}+{\tilde{J}}_{R}^{†}{\hat{u}}_{R}\right\|}_{\infty }^{-1},$$

where we consider that the linear velocity is synchronized with the angular velocity, thus $\Omega_{\max}=h^{-1}\cdot V_{\max}$ and $V_{\max}=h\cdot \Omega_{\max}$. In contrast to the existing methods, the proposed DTF method gives us the physically meaningful and accurate information about how fast the robot can move when a simultaneous translational and rotational motion is needed in directions ${\hat{u}}_{T}$ and ${\hat{u}}_{R}$, respectively. The maximum linear (angular) velocity vector, considering the desired angular (linear) motion constraint, can be obtained as follows:

(32) $$v_{\max}=V_{\max}{\hat{u}}_{T},$$

(33) $$\omega_{\max}=\Omega_{\max}{\hat{u}}_{R},$$

which can be combined further into the maximum feasible twist vector $t_{\max}^{T}=\left[\begin{array}{cc} v_{\max}^{T} & \omega_{\max}^{T} \end{array}\right]$ for the given base task parameters (${\hat{u}}_{T}$, ${\hat{u}}_{R}$, h) and under the constraints (8).

**Figure 4:**
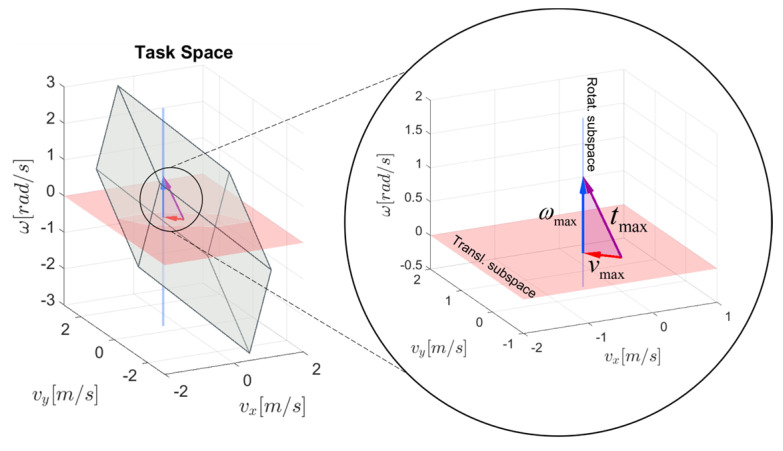
Graphical representation of the proposed DTF method with the twist manipulability polytope $P^{twist}$ (**left**), and in a detailed view (**right**).

To provide an intuitive understanding of the proposed DTF method, the maximum linear and angular velocity vectors ($v_{\max}$ and $\omega_{\max}$) are shown graphically by the twist manipulability polytope in Figure 4. In the case of the 3 DOF planar robotic mechanism, the twist manipulability polytope lies in the 3-dimensional space, which consists of the 2-dimensional translational subspace (the $v_{x}-v_{y}$ plane) and the 1-dimensional rotational subspace (ω-axis). The maximum linear velocity capability is illustrated as a vector $v_{\max}$ in red, with the direction ${\hat{u}}_{T}$ and the magnitude $V_{\max}$, which lie in the $v_{x}-v_{y}$ plane, and it can be interpreted as the orthogonal projection of the twist vector onto the translational subspace. The maximum angular velocity capability is shown as a vector $\omega_{\max}$ in blue in the ω-axis, with the direction ${\hat{u}}_{R}$ and the magnitude $\Omega_{\max}$. The tip of the resultant twist vector $t_{\max}$ in purple is touching the surface of the twist manipulability polytope.

**Figure 5:**
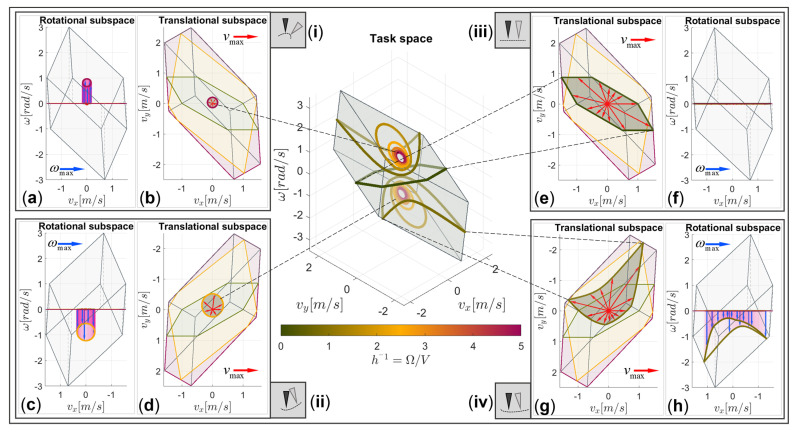
Twist polytope $P^{twist}$ with the iso-curves of the same task requirements (*h* = const.); (**a**,**c**,**f**,**h**) a polytope-based representation of the feasible angular velocity ($\omega_{\max}$ —blue) for (i) a concave path segment, (ii) a convex path segment, (iii) a flat path segment, and (iv) a flat convex path segment, respectively; (**b**,**d**,**e**,**g**) a polytope-based representation of the feasible linear velocity ($v_{\max}$ —red) for (i) a concave path segment, (ii) a convex path segment, (iii) a flat path segment, and (iv) a flat convex path segment, respectively.

Unlike the existing methods [32,49] that separately evaluate maximal end-effector capabilities based on the desired velocity direction for the translational and the rotational subspaces, the proposed DTF method, although based on twist decomposition, links both individual subspace constraints by the inclusion of the specific robot task requirements with the velocity ratio factor *h*. In terms of the robot machining of the workpiece with complex surface geometric properties, the task requirements depend on the variation in the surface curvature and the defined tool path, which is then reflected in the velocity ratio factor *h*. Thus, for a given translational direction ${\hat{u}}_{T}$, rotational direction ${\hat{u}}_{R}$, and velocity ratio factor *h*, we seek the maximum linear $V_{\max}$ and rotational velocity $\Omega_{\max}$ that could be achieved. To provide an illustrative and intuitive demonstration of the proposed DTF method with respect to different task requirements, the maximal linear and angular end-effector velocities are illustrated graphically by the task-space twist polytope in Figure 5.

In the middle of Figure 5, we show the twist polytope with the curves on its surface, described by the tips of the resultant twist velocity vectors at different values of the velocity ratio factor *h,* while the translational direction vector interpolates as ${\hat{u}}_{T}=\left[\begin{array}{cc} \cos(\alpha ) & \sin(\alpha \end{array})\right]$, α=0…2π. Note that the different values of the velocity ratio factor *h* are related to different task requirements, which, in our case, are chosen as: (i) Concave path segment with *h* = 0.21, (ii) Convex path segment with *h* = 0.42, (iii) Flat path segment with *h* = 1000, (iv) Flat convex path segment with *h*=1.25. Each of the polytope iso-curves is related to the evaluation of the twist vector tips with the same task requirements at the specific value of the synchronization parameter *h* (also see the colorbar for the selected value). It can be noted that each iso-curve lies entirely on the surface of the manipulability polytope, thus satisfying the constraint given by (8). If the reverse direction of angular velocity is considered, the set of resultant tips are mirrored to the opposite side of the twist manipulability polytope.

The maximum linear and angular velocity vectors of each example (i)–(iv) is additionally shown in detail by two different projection views, i.e., the $v_{x}-\omega$ plane and the $v_{x}-v_{y}$ plane, respectively. In the $v_{x}-v_{y}$ plane, the maximum feasible linear velocity vectors (plotted in red) can be obtained if the unit direction vector ${\hat{u}}_{T}$ is interpolated as described above. Note that this projection view also depicts the translational polytopes $P_{\infty }^{T}$, $P_{2}^{T}$, and $P_{strong}^{T}$ in reddish, greenish, and yellowish colors, respectively. The corresponding angular velocity vectors are plotted in blue in the $v_{x}-\omega$ plane for the directions ${\hat{u}}_{R}=+1$ or ${\hat{u}}_{R}=-1$. The maximum feasible magnitude of the linear and angular velocity vectors for the same combination of ${\hat{u}}_{T}$ and ${\hat{u}}_{R}$ obviously depends on the value of the synchronization parameter h.

The example (i) represents the case where the curvature of the path is high, and the change in the end-effector orientation is much bigger than the change in position (the highest value of $h^{-1}$). Since the example path curve is of a concave function shape, the direction of angular velocity is ${\hat{u}}_{R}=+1$, and the tips of the resultant vectors are located on the upper part of the twist manipulability polytope $P^{twist}$(see Figure 5a)). The set of maximum feasible linear velocity vectors form a circle; thus, the maximum directional kinematic capabilities are the same in all directions (see Figure 5b). Since almost only angular motion is needed in this case, the maximum feasible linear velocities based on the proposed DTF method ($v_{\max}$) are quite low. Note that in the case of the translational manipulability polytope $P_{\infty }^{T}$ (reddish) or $P_{2}^{T}$ (yellowish), the maximum linear velocity vector would end on the surface of each polytope (see Figure 5b) since the analysis of linear velocity capabilities are evaluated separately from the rotational subspace in this case.

An example of the path segment with a convex function shape (${\hat{u}}_{R}=-1$) is demonstrated in the case (ii). The tips of the resultant vectors are located at the bottom of the manipulability polytope (see Figure 5c) in this case. The desired combination of linear and angular motion can be performed faster in the +y direction than in the −y direction if the same direction of angular velocity is considered (see Figure 5d). The magnitude of the maximum feasible linear velocity based on the manipulability polytope $P_{\infty }^{T}$ (reddish) is the same as in Figure 5b; an even more flattened path segment is considered in this case, and different synchronization between the linear and angular velocities is needed (more linear motion).

In the example (iii), almost only translational motion is considered. The magnitudes of the linear velocity vectors are equal to the vector lengths of the translational manipulability polytope in the strong sense $P_{strong}^{T}$ (see Figure 5e). The resultant vectors lie in the $v_{x}-v_{y}$ plane since there is no rotational motion (see Figure 5f). Although the results based on the manipulability polytope $P_{strong}^{T}$ (greenish) can give the same solution as the proposed method, the analysis of this polytope is not suitable for other cases, where synchronization between the linear and angular motion is needed.

In contrast to (iii), example (iv) represents a more flattened path segment. The magnitudes of the feasible linear velocity vectors are larger than the magnitudes of the angular velocity vectors. Especially in Figure 5g,h, it can clearly be seen that the maximal directional kinematic capabilities are strongly dependent on the direction of the linear velocity vector ${\hat{u}}_{T}$, considering that the velocity ratio factor *h* and the direction of angular velocity ${\hat{u}}_{R}$ for all directions are the same. As it can be seen from Figure 5g, the geometry obtained by the proposed DTF method touches the translational manipulability polytope $P_{\infty }^{T}$ (reddish) only in two directions, which means that only for those directions, the same solution can be obtained based on the translational manipulability polytope in the weak sense. For all other directions, the linear velocity evaluated by $P_{\infty }^{T}$ can be feasible only if suitable synchronization to rotational motion is considered, as can be seen in Figure 5h. The angular velocity vector should not exceed the dimension of the twist manipulability polytope.

Note that Figure 5b,d,e,g beside the weak and the strong translational polytopes show regions of feasible linear velocities under the constraint of the task-dependent velocity ratio factor *h,* which are introduced as subregions within the $L_{\infty }$ weak polytopes.

## 3. Results

To demonstrate the effectiveness and potential usefulness of the proposed DTF method, we present two case studies in which the maximal end-effector capabilities were evaluated, i.e., for a generic 3DOF planar robot manipulator and for the 6DOF collaborative robot UR5e. To ensure the physical consistency of such evaluation in the case of a robot machining task, where the simultaneous control of synchronous translational and rotational motion is required, the maximal linear and angular velocity of the end-effector will be given individually for a predefined path segment. We will show that the maximal end-effector capabilities do not only depend on the configuration and direction of the desired linear and angular motion, but they are also strongly dependent on the specific task requirements. Additionally, in the case of the collaborative robot UR5e, it will be shown that this criterion could be useful in the case of a task-oriented workspace analysis and an optimal task placement.

### 3.1. Basic Case Study Formulation

We consider the robot machining trajectory along a complex curved surface that is divided into N equally spaced curve segments defined by M=N+1 waypoints. For this evaluation, the maximum end-effector kinematic capabilities will be analyzed to move the robot end-effector from an initial point to a target point in each segment, while aiming not to exceed the joint velocity limits. In addition, the end-effector tip should follow the desired surface along the path all the time, moving with constant linear velocity and synchronizing the angular velocity to maintain the tool orientation normal to the surface. Our goal was to calculate the maximum feasible linear velocity for each segment of the desired path. In this study, three different parts of the geometrically curved surface will be analyzed and used to demonstrate the proposed DTF method graphically and numerically.

### 3.2. Generic 3 DOF Planar Manipulator

#### Path Segment Feasiblity

**Figure 6:**
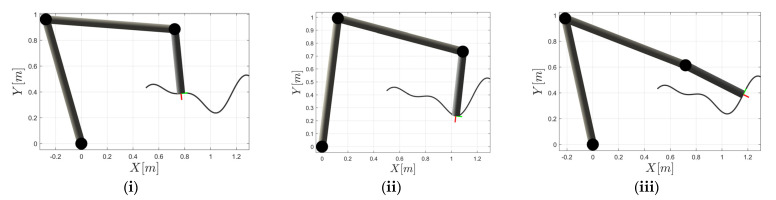
Three different parts of the curved path: (**i**) Slightly curved path segment; (**ii**) Strongly curved path segment; (**iii**) Flat path segment.

The proposed DTF method was firstly applied to a generic 3 DOF planar manipulator, which enables an easier graphical representation of the manipulability polytope in a twist space. The 2D curve shown in Figure 6 represents the path that the manipulator end-effector followed. A set of waypoints was placed along the path, with the orientation normal to the curve. The end-effector was to simultaneously reach each of them at the desired position and orientation. In this experiment, the proposed DTF method was used to determine the maximum feasible linear velocity at three different parts of the curved path, as shown in Figure 6. Example (i) represents a slightly curved path segment, whereas a strongly curved path segment was considered in example (ii), and a flat path segment was analyzed in example (iii). We assume that the maximum kinematic capabilities of the robot are limited by the joint velocity ${\dot{\theta }}_{\max}=1\;(\mathrm{rad}/s)$ for all joints.

At each of the path segments under consideration, the direction of linear and angular velocity was determined along the path. Since simultaneous synchronous linear and angular motion was required along the path, the relative importance of each motion type plays a crucial role in the accurate determination of the maximal end-effector capabilities. In the case of following the curved path, the ratio between the translations and the rotations depends on its curvature. During the motion along the strongly curved path segment, as in case (ii), a significant change in the end-effector orientation was required, while a relatively small change in position was performed; therefore, the ratio between the magnitudes of the linear and angular velocities, denoted by the synchronization parameter *h*, converged to 0. For the flat path segment (iii), the synchronization parameter *h* value was the highest since the linear motion dominated over the angular. The values for the considered TCP position, the direction of the linear velocity vector ${\hat{u}}_{T}$, the direction of the angular velocity vector ${\hat{u}}_{R}$, and the velocity ratio factor *h* of each path segment are summarized in Table 1.

Based on the proposed DTF method, the maximum feasible directional kinematic capabilities ($V_{\max}$ denotes the maximal magnitude of linear velocity evaluated by (30), whereas $\Omega_{\max}$ denotes the maximal magnitude of angular velocity evaluated by (31)) was calculated for each path segment. The required joint velocities were also computed to confirm that condition (8) was satisfied. From Table 2, it can be noted that the computed combination of the maximal linear and angular velocity reaches the joint velocity limit in at least one joint in all cases. Although the velocity limit was achieved in all path segments, at least at a single joint, the maximum linear velocity varied, depending on the considered robot configuration, the linear and angular velocity directions, and the specific task requirements linked to the curvature of the path that determines the velocity ratio factor *h*.

**Figure 7:**
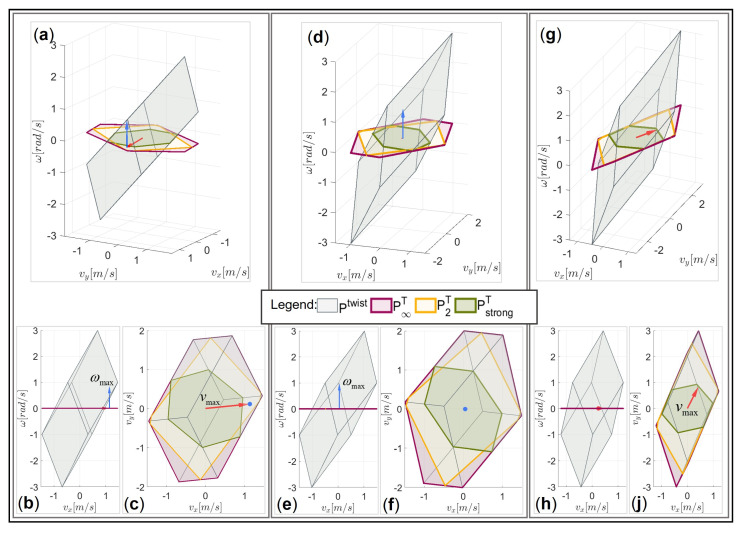
Graphical representation of *v*_max_ and $\omega_{\max}^{}$ by the DTF method for a generic 3DOF planar robot manipulator in relation to its manipulability polytopes $P^{twist}$, $P_{\infty }^{T}$, and $P_{strong}^{T}$, respectively, for segment (i): (**a**) the 3D projection view; (**b**) the $v_{x}-\omega$ plane view; (**c**) the $v_{x}-v_{y}$ plane view; segment (ii): (**d**) the 3D projection view; (**e**) the $v_{x}-\omega$ plane view; (**f**) the $v_{x}-v_{y}$ plane view; segment (iii): (**g**) the 3D projection view; (**h**) the $v_{x}-\omega$ plane view; (**j**) the $v_{x}-v_{y}$ plane view.

The highest maximum feasible linear velocity was achieved in the case of segment (i), where the required change in position was approximately the same in relation to the change in the orientation (h≈1). The lowest maximum feasible linear velocity was obtained for segment (ii), where almost only the angular motion was needed. For a better understanding of the computed results, the graphical representation of the proposed DTF method is presented in Figure 7 in relation to the manipulability polytopes defined above.

For each of the considered examples (i), (ii), and (iii), the twist polytope $P^{twist}$ and the translational manipulability polytopes $P_{\infty }^{T}$, $P_{2}^{T}$, and $P_{strong}^{T}$ are illustrated in Figure 7 in three different views, i.e., the 3D projection view, the 2D view in the $v_{x}-\omega$ plane, and the 2D view in the $v_{x}-v_{y}$ plane, respectively. The figure also depicts the maximum feasible linear and angular velocities, as determined by the proposed DTF method given by (30), (31).

It can easily be verified that the translational manipulability polytope $P_{\infty }^{T}$ fits to the orthogonal projection of the twist manipulability polytope $P^{twist}$ onto the translational subspace, shown by the $v_{x}$–$v_{y}$ plane in this case (Figure 7c,f,j). For the synchronous linear and angular motion, considering the desired task requirements given by the synchronization parameter $v_{\max}^{}$ *h*, the maximal linear velocity vector $v_{\max}^{}$ (see (32)) based on the proposed DTF method was calculated and shown as a vector in red. For the desired motion considered for the segment (i), the magnitude of the maximum linear velocity vector $v_{\max}^{}$ was smaller than the vector length of the manipulability polytope $P_{\infty }^{T}$. When almost only angular motion was needed, as in the case of segment (ii), the maximum linear velocity $v_{\max}^{}$ was near zero. In the case of segment (iii), where the linear motion dominates the angular motion, the value of $V_{\max}^{}$ was equal to the vector length of the translational manipulability polytope $P_{strong}^{T}$ in the strong sense, which was also the highest feasible linear velocity for this combination of ${\hat{u}}_{T}$ and ${\hat{u}}_{R}$.

The $v_{x}-\omega$ plane view (Figure 7b,c,e) shows the maximum feasible angular velocity capabilities of each path segment. The maximum feasible angular velocity for the specific task requirements of each considered segment is shown as a vector $\omega_{\max}$ in blue. The maximum feasible angular velocity can be achieved for segment (ii), where the angular motion dominates the linear motion. A relatively high angular velocity can also be performed for the synchronous movement considered for segment (ii), while the lowest angular velocity can be performed in the case of segment (iii) since almost only linear motion was needed.

### 3.3. Collaborative Robot UR5e

#### 3.3.1. Path Segment Feasiblity

**Figure 8:**
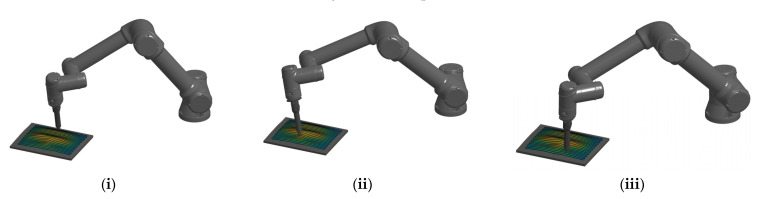
Collaborative robot UR5e with three different parts of the curved surface: (**i**) a flat path segment; (**ii**) a strongly curved path segment; (**iii**) a slightly curved path segment.

The proposed DTF method was also demonstrated and verified on the collaborative robot UR5e, which is of 6DOF, thus enabling robot operation in a 3D space. In this case, the 3D curve on the workpiece surface was determined as the path that the robot tool had to follow. A set of waypoints was placed along the path, with the orientation normal to the surface. The robot end-effector was to simultaneously reach each of them with the desired position and orientation. In this experiment, the proposed DTF method will be used to determine the maximum feasible linear velocity at three different parts of the curved path, as shown in Figure 8, if we assume that the maximum kinematic capabilities of the robot are limited by the joint velocity ${\dot{\theta }}_{\max}=3.14\;\mathrm{rad}/s$ for all joints. Similar to the 3 DOF planar case, example (i) represents a flat path segment of the surface, a strongly curved path segment was considered in example (ii), and a slightly curved path segment of the surface was analyzed in example (iii).

At each of the segments under consideration, the directions of the linear and angular velocities were determined along the path. During the motion along path segment (ii), a significant change in the end-effector orientation was required, while a relatively small change in position was performed; therefore, the ratio between the magnitudes of the linear and angular velocities, denoted by h, converged to 0. On the relatively flat path segment (i), the velocity ratio factor h was the highest since the linear motion dominated the angular motion. The direction of the linear velocity vector ${\hat{u}}_{T}$, the direction of the angular velocity vector ${\hat{u}}_{R}$, and the velocity ratio factor *h* of each segment are shown in Table 3.

The maximum linear velocity $V_{\max}$ and maximum angular velocity $\Omega_{\max}$ based on the DTF method are shown in Table 4, together with the required joint velocities $\dot{\theta }$.

**Figure 9:**
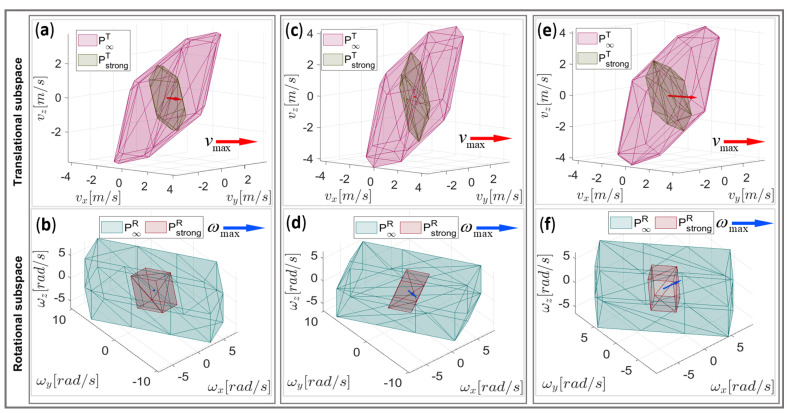
Graphical representation of *v*_max_ and $\omega_{\max}$ and determined by the DTF method for the 6DOF UR5e robot manipulator in relation to its manipulability polytopes within the translational subspace $P_{\infty }^{T}$, $P_{strong}^{T}$, and the rotational subspace $P_{\infty }^{R}$ and $P_{strong}^{R}$, respectively, for segment (i): (**a**) $v_{\max}$ and (**b**) $\omega_{\max}$; for segment (ii): (**c**) $v_{\max}$ and (**d**) $\omega_{\max}$; and for segment (iii): (**e**) $v_{\max}$ and (**f**) $\omega_{\max}$.

For each of the considered examples (i), (ii), and (iii), the 3D translational manipulability polytope $P_{\infty }^{T}$ in the $L_{\infty }$ weak sense (magenta), the 3D translational manipulability polytope $P_{strong}^{T}$ in the strong sense (green), the 3D rotational manipulability polytope $P_{\infty }^{R}$ in the $L_{\infty }$ weak sense (blue), and the 3D rotational manipulability polytope $P_{strong}^{R}$ in the strong sense (red) are all illustrated in Figure 9. The maximum feasible linear velocity vector $v_{\max}$ (red arrow) and the maximum feasible angular velocity vector $\omega_{\max}$ (blue arrow) by the proposed DTF method are also illustrated. Note that the twist space in this case is of 6D, and thus inconvenient for a graphical presentation of the 6D twist polytope $P^{twist}$.

If the main concern was the linear velocity (the value of the synchronization parameter h was high), as in case (i), the maximal linear velocity vector $v_{\max}^{}$ in the direction ${\hat{u}}_{T}$ practically coincides with the vector length of the translational manipulability polytope $P_{strong}^{T}$ in the strong sense in the same direction. The maximal angular velocity $\Omega_{\max}^{}$ was near zero since the flattest part of the surface was considered. On the other hand, the lowest value of the synchronization parameter h was when a greater change in orientation was required as compared to the change in position, as it was in case (ii). As a result, the maximal angular velocity $\Omega_{\max}^{}$ was, in this case, equal to the vector length of the rotational manipulability polytope $P_{strong}^{R}$ in the strong sense, and the maximal feasible linear velocity $V_{\max}^{}$ was the lowest among all three cases. When the synchronized linear and angular motion was taken into account, as in case (iii), the maximum linear and angular velocities were higher, and its vectors exceeded the bounds of the translational (rotational) manipulability polytope $P_{strong}^{T}$ ($P_{strong}^{R}$) in the strong sense. Generally, however, they can never exceed the bounds of the translational (rotational) manipulability polytope $P_{\infty }^{T}$ ($P_{\infty }^{R}$) in the weak sense.

#### 3.3.2. Workspace Analysis

**Figure 10:**
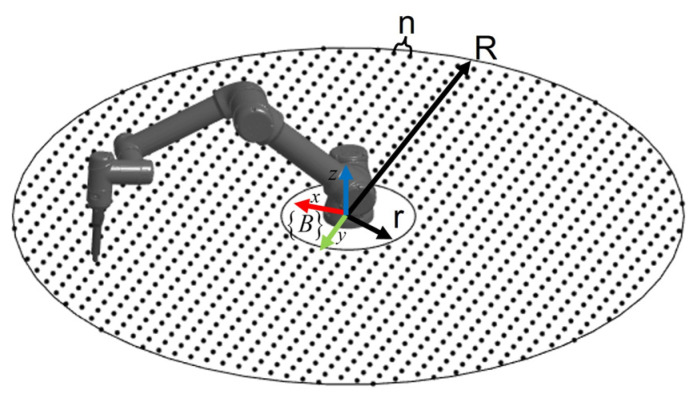
Discretization of the robot workspace.

An important issue in robot task design is to place the robot tool path in such a location in the robot’s workspace where the robot will be capable of performing a desired movement utilizing the lowest possible joint velocities, or, in other words, performing a desired movement with maximum feasible tool velocity. In order to demonstrate that we can apply the proposed DTF method, we generated a colored task-oriented kinematic capability map, which represents the maximum directional kinematic capabilities for the desired movement. The kinematic capabilities were computed for equidistantly distributed sets of points in the UR5e’s horizontal x-y plane (z = 0) of the base robot frame {B}, as shown in Figure 10, where *n* = 0.05 m denotes the discretization step for both directions, and *R* = 1 m and *r* = 0.2 m represent the inner and the outer workspace boundaries, respectively. The maximum linear velocity was calculated at each discretized point along a specified task direction (i), (ii), and (iii) (see Figure 8), which required synchronous translational–rotational motion. The robot configuration was chosen, as shown in Figure 10, for all discretized points.

**Figure 11:**
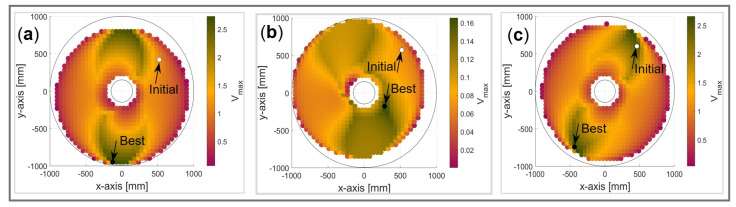
The task-oriented kinematic capability map based on the DTF method for: (**a**) Segment (i); (**b**) Segment (ii); (**c**) Segment (iii).

At each point, the inverse kinematic solution was firstly computed for the considered robot configuration. Only those points for which the inverse kinematic solution exists were included in further workspace analysis; the others were disregarded. The maximum linear velocity $V_{\max}$ based on the proposed DTF method (30) was calculated for those with a valid inverse kinematic solution; then each of them was assigned to a different color, ranging from magenta to green (see the colorbar in Figure 11 for the selected value), which formed a so-called task-oriented kinematic capability map, illustrated in Figure 11 for each path segment. The magenta-colored areas represent the placement with the lowest feasible linear velocity (the worst robot kinematic capabilities for the desired task), and the green-colored areas represent locations with the maximum value of the feasible linear velocity (the best robot kinematic capabilities for the desired task). Since $V_{\max}$ is related to $\Omega_{\max}$ by $\Omega_{\max}=h^{-1}\cdot V_{\max}$, the distribution of the colormap would be the same for $\Omega_{\max}$, only scaled by $h^{-1}$. The randomly chosen initial placements of each considered segment (see Figure 8) are denoted by the white point on the generated task-oriented kinematic capability maps, while the locations with the highest maximum linear velocity $V_{\max}$ are represented by the black point.

As shown in Figure 11, the distribution of the maximum linear velocity $V_{\max}$ based on the proposed DTF method varied greatly according to the task requirements. Although the considered direction of the linear velocity vector ${\hat{u}}_{T}$ was pointing in almost the same direction in the case of path segments (i) and (iii) (see Table 3 and Figure 9), the best locations to perform a required movement were not located in the same place in the robot workspace since the requirements for rotational movement were quite different. An even greater difference in the distribution of the maximum linear velocity can be seen for path segment (ii), where the angular motion dominated the linear.

The feasible directional kinematic capabilities for the best placement of each path segment are presented in Table 5. In comparison to the maximum kinematic capabilities from Table 4, much higher linear velocity can be achieved under the same joint velocity constraints (8).

By the optimal placement of the workpiece with the considered machining path, a great improvement in the linear velocity amplitude can be achieved, while keeping the same desired base task parameters (${\hat{u}}_{T}$, ${\hat{u}}_{R}$, h). The improvement of the maximum linear velocity for all three path segments are shown in percentages in Table 6.

**Figure 12:**
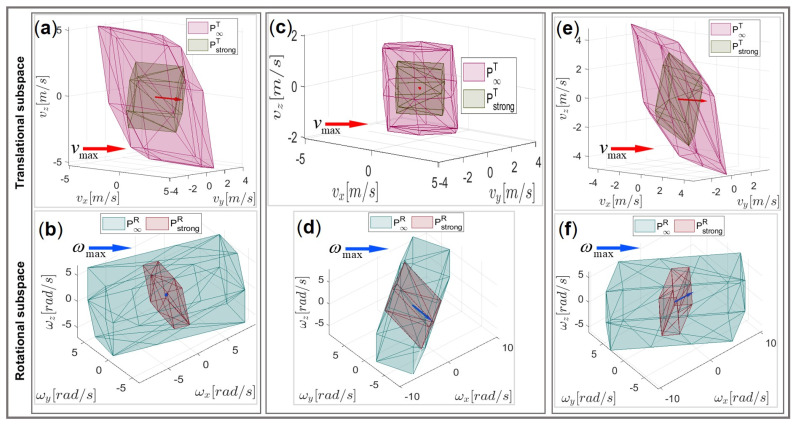
Graphical representation of $v_{\max}$ and $\omega_{\max}$ for the best path segment placement, determined by the DTF method for the 6DOF UR5e robot manipulator in relation to its manipulability polytopes within the translational subspaces $P_{\infty }^{T}$, $P_{strong}^{T}$, and rotational subspaces $P_{\infty }^{R}$ and $P_{strong}^{R}$, respectively, for segment (i): (**a**) $v_{\max}$ and (**b**) $\omega_{\max}$; for segment (ii): (**c**) $v_{\max}$ and (**d**) $\omega_{\max}$; and for segment (iii): (**e**) $v_{\max}$ and (**f**) $\omega_{\max}$.

Similar to Figure 9, the maximum linear and angular velocity vectors based on the proposed DTF method are shown with respect to the translational and rotational manipulability polytope in both the weak and the strong senses, respectively, in Figure 12. The shape of each polytope and the amplitude of each vector were quite different in comparison to the initial placement of the task segments, and even if the considered task requirements (${\hat{u}}_{T}$, ${\hat{u}}_{R}$, h) were the same.

## 4. Discussion and Conclusions

When planning a feasible robotic task, the required joint capabilities should be lower than the maximum capabilities available. To evaluate the feasible kinematic directional capabilities for robot machining, an approach called the Decomposed Twist Feasibility (DTF) method has been proposed in this paper. The basic idea behind the proposed DTF method is to find the maximal feasible directional kinematic capabilities in an operational space, individually for translational and rotational performances, under the robot joint velocity constraints. The method was algebraically derived and described by the manipulability polytopes as well. In contrast to the conventional manipulability methods, the proposed DTF method can provide accurate and dimensionally homogeneous information on how fast the robot end-effector can move under the constraint of joint velocities while maintaining the base task requirements. It can give us reliable information about the maximum linear and angular velocities when synchronized translational/rotational motion is required. A graphical representation of the proposed DTF method was presented by the twist manipulability polytope in the case of a generic 3DOF planar mechanism. Although we gave individual consideration to the translational and rotational capabilities in the proposed DTF method, the presented results show that the tip of the resulting maximum feasible twist velocity vector obtained by (30)–(33) always ends on the surface of the well-known twist manipulability polytope at the given joint velocity constraint (8), which verifies the method. In comparison to the conventional analysis of the twist manipulability polytope—which requires a computation of its vertices, the construction of the corresponding convex hull, and a further computation analysis of the convex hull in order to determine the intersection point with the polytope surface in the desired direction—the computational complexity can be reduced significantly by the DTF method, and the accuracy of the resulting information is maintained as well. The usefulness was demonstrated, especially in the case of the robot machining task purpose on a generic 3DOF planar mechanism and the collaborative robot UR5e. The maximum feasible linear velocity, considering the desired rotational motion, was determined in order to follow a specific path segment on the workpiece surface. The regions with the highest and lowest maximum feasible linear velocities were determined and then illustrated by the colormap, which forms the so-called task-oriented kinematic capability map.

In conclusion it should be noted that the proposed DTF method addresses a purely kinematic performance measure. To provide feasible and optimal workpiece placement, the dynamic performances regarding the given task parameters should also be considered. In order to find an optimal workpiece placement for the entire tool path, the maximum linear velocity should be calculated for each path segment, or at least for the most critical ones. Thus, the proposed DTF method could be used as a suitable optimization criterion for optimal task-oriented workpiece placement within the robot’s workspace.

## Figures and Tables

**Table 1:** Task requirements for three different path segments (3 DOF manipulator).

Path Segment	Position	${\hat{u}}_{T}$	${\hat{u}}_{R}$	h
(i)	X: 0.7720, Y: 0.3882	$\left[\begin{array}{cc} 0.7720 & 0.3882 \end{array}\right]$	$\left[+1\right]$	1.3231
(ii)	X: 1.0340, Y: 0.2374	$\left[\begin{array}{cc} 1.0340 & 0.2374 \end{array}\right]$	$\left[+1\right]$	0.0493
(iii)	X: 1.1640, Y: 0.3877	$\left[\begin{array}{cc} 1.1640 & 0.3877 \end{array}\right]$	$\left[-1\right]$	62.4704

**Table 2:** Feasible directional kinematic capabilities for three different path segments (3 DOF manipulator).

Path Segment	$V_{\max}(m/s)$	$\Omega_{\max}(\mathrm{rad}/s)$	$\dot{\theta }(\mathrm{rad}/s)$
(i)	1.1439	0.8646	$\left[\begin{array}{ccc} -0.7471 & 0.6117 & 1.0000 \end{array}\right]$
(ii)	0.0489	0.9935	$\left[\begin{array}{ccc} 0.4469 & -0.4534 & 1.0000 \end{array}\right]$
(iii)	0.9471	0.0152	$\left[\begin{array}{ccc} -0.1118 & 1.0000 & -0.9034 \end{array}\right]$

**Table 3:** Task requirements for three different path segments (UR5e).

Path Segment	${\hat{u}}_{T}$	${\hat{u}}_{R}$	h
(i)	${\left[\begin{array}{ccc} 0.9999 & 0 & 0.0117 \end{array}\right]}^{T}$	${\left[\begin{array}{ccc} 0.6209 & 0.7625 & -0.1820 \end{array}\right]}^{T}$	4.4632
(ii)	${\left[\begin{array}{ccc} -0.9893 & 0 & 0.1459 \end{array}\right]}^{T}$	${\left[\begin{array}{ccc} 0.0865 & -0.9761 & -0.1994 \end{array}\right]}^{T}$	0.0360
(iii)	${\left[\begin{array}{ccc} 0.9992 & 0 & 0.0411 \end{array}\right]}^{T}$	${\left[\begin{array}{ccc} 0.9997 & -0.0249 & 0.0011 \end{array}\right]}^{T}$	0.7454

**Table 4:** Feasible directional kinematic capabilities for three different path segments (UR5e).

Path Segment	$V_{\max}(m/s)$	$\Omega_{\max}(\mathrm{rad}/s)$	$\dot{\theta }\left(\mathrm{rad}/s\right)$
(i)	1.1552	0.2588	${\left[-0.9854,\;1.7508,\;-3.1416,\;1.2716,\;0.1298,\;-0.9807\right]}^{T}$
(ii)	0.0699	1.9391	${\left[0.3208,-1.2202,\;2.4198,\;-3.1416,\;-0.7500,\;0.8459\right]}^{T}$
(iii)	2.4365	3.2688	${\left[-3.1063,\;1.6537,\;-2.7480,\;-1.4495,\;2.2663,\;-3.1416\right]}^{T}$

**Table 5:** Feasible directional kinematic capabilities for three different path segments—best placement.

**Path Segment**	$V_{\max}(m/s)$	$\Omega_{\max}(\mathrm{rad}/s)$	$\dot{\theta }(\mathrm{rad}/s)$
(i)	2.7351	0.6128	${\left[3.1416,-1.6230,\;3.1328,\;-1.5284,\;-1.2614,\;3.0600\right]}^{T}$
(ii)	0.1656	4.5959	${\left[-3.0173,-2.9210,\;2.8698,\;-2.7092,\;2.9541,\;-3.1416\right]}^{T}$
(iii)	2.6605	3.5693	${\left[3.1416,-2.0760,\;2.9403,\;1.6904,-2.3072,\;3.1057\right]}^{T}$

**Table 6:** Comparison between the maximum linear velocity for the initial and best placements.

Path Segment	$V_{\max}(m/s)—\mathrm{Initial}\;\mathrm{Placement}$	$\Omega_{\max}(\mathrm{rad}/s)—\mathrm{Best}\;\mathrm{Placement}$	%
(i)	1.1552	2.7351	136.77
(ii)	0.0699	0.1656	136.91
(iii)	2.4365	2.6605	9.19

## Data Availability

Not applicable.
